# Resistance and Tolerance to Imperfectly Specialized Parasites: Milkweed Butterflies and Their Protozoan Parasites

**DOI:** 10.1002/ece3.70979

**Published:** 2025-03-04

**Authors:** Maria L. Müller‐Theissen, Nicole L. Gottdenker, Sonia M. Altizer

**Affiliations:** ^1^ Odum School of Ecology University of Georgia Athens GA USA; ^2^ Center for the Ecology of Infectious Diseases University of Georgia Athens GA USA; ^3^ Department of Pathology College of Veterinary Medicine, University of Georgia Athens GA USA

**Keywords:** cross‐infection, *Danaus gilippus*, *Danaus plexippus*, host specificity, monarch butterfly, neogregarine, *Ophryocystis elektroscirrha*, queen butterfly

## Abstract

Understanding host specificity and cross‐species transmission of parasites is crucial for predicting the risk and consequences of parasite spillover. We experimentally examined these dynamics in two closely related, sympatric, milkweed butterfly hosts: monarchs ( *Danaus plexippus* ) and queens ( *D. gilippus* ). The debilitating protozoan *Ophryocystis elektroscirrha* (*OE*) infects wild monarchs throughout their range, and similar neogregarine parasites have been reported from queens. We compared host resistance and tolerance to infection between hosts exposed to parasites of conspecific and heterospecific origin and examined whether differences in immune investment reflected variation in infection outcomes. Results showed that monarchs were highly susceptible to both conspecific and heterospecific parasites. In contrast, queens were susceptible almost exclusively to conspecific parasites. Queens showed greater tolerance to infection and greater immune defense in the form of melanization activity and concentration of encapsulating hemocytes. Additionally, monarch parasites caused higher pre‐adult mortality and more wing deformities than queen parasites. Given that *OE* can reduce monarch abundance and migratory performance, quantifying cross‐infection outcomes is important for conservation management of these two butterfly species. The greater susceptibility and costs of infection in monarchs suggest potential fitness trade‐offs against resistance and tolerance to infection in migratory hosts and underscore the need to identify factors that limit hosts' adaptation to parasites.

## Graphical abstract

This study explores the causes and outcomes of cross‐species parasite transmission, focusing on two congeneric and sympatric butterfly host species: monarchs (*Danaus plexippus*) and queens (*D. gilippus*), and their respective parasite strains (*Ophryocystis elektroscirrha* and similar parasites). Monarchs were highly susceptible to both conspecific and heterospecific parasites, whereas queens showed greater resistance, tolerance, and immune defense, underscoring the importance of quantifying cross‐infection outcomes to inform conservation strategies for these two butterfly species.

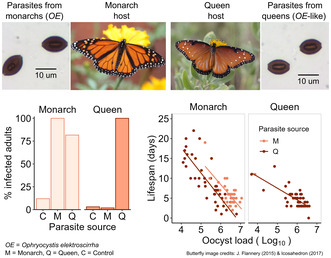

## 1 Introduction

Most parasites infect multiple host species (Cleaveland et al. 2001). These multi‐host parasites often infect geographically overlapping, closely related, and ecologically similar hosts that provide increased opportunities for cross‐species exposure and colonization (Huang et al. 2014; Stephens et al. 2019; Streicker et al. 2010). This is partly because phylogenetically related hosts may exhibit similar physical, physiological, immunological, and molecular traits that facilitate parasite colonization (Gilbert and Webb 2007; Longdon et al. 2014). However, parasite shifts between distantly related hosts can also occur when cross‐species exposures are frequent, such as when multiple host species share habitat patches and resources (Campisano et al. 2014). Importantly, multi‐host parasites differ in their ability to exploit different host species effectively, and host species differ in their susceptibility to distinct strains or isolates of otherwise similar parasites (Antonovics et al. 2013; Lievens et al. 2018).

Host resistance, defined as the ability to block infection or slow parasite development, depends on both host and parasite properties, making it challenging to disentangle the factors driving infection outcomes (Antonovics et al. 2013). A parasite's degree of specialization to its host can similarly depend on several factors, including host availability and behavior, biochemical cues, tissue or cell receptivity, and effects on host morbidity or mortality that determine the infectious period (Woolhouse et al. 2001). Frequent host and parasite interactions and costs of infection for hosts are expected to select for host‐evolved resistance and adaptations of parasites to overcome host defenses (Buckling and Rainey 2002; Kaltz and Shykoff 1998). Thus, parasite specialization and host–parasite coevolution can result in closely related parasites differing in their ability to exploit different numbers and identities of host species (Bruns et al. 2021). Past studies on viral, bacterial, and fungal pathogens in humans and plants have explored mechanisms of host specificity, particularly those involving molecular interactions between the pathogens and their hosts [e.g., (Best et al. 2010; Pan et al. 2014; Li et al. 2020)]. However, more empirical studies are needed to understand host specificity in insect systems, which is crucial for managing pest species and predicting parasite threats to pollinators and insects of conservation concern (Bartel and Altizer 2012; Fürst et al. 2014; Manley et al. 2015).

**FIGURE 1:**
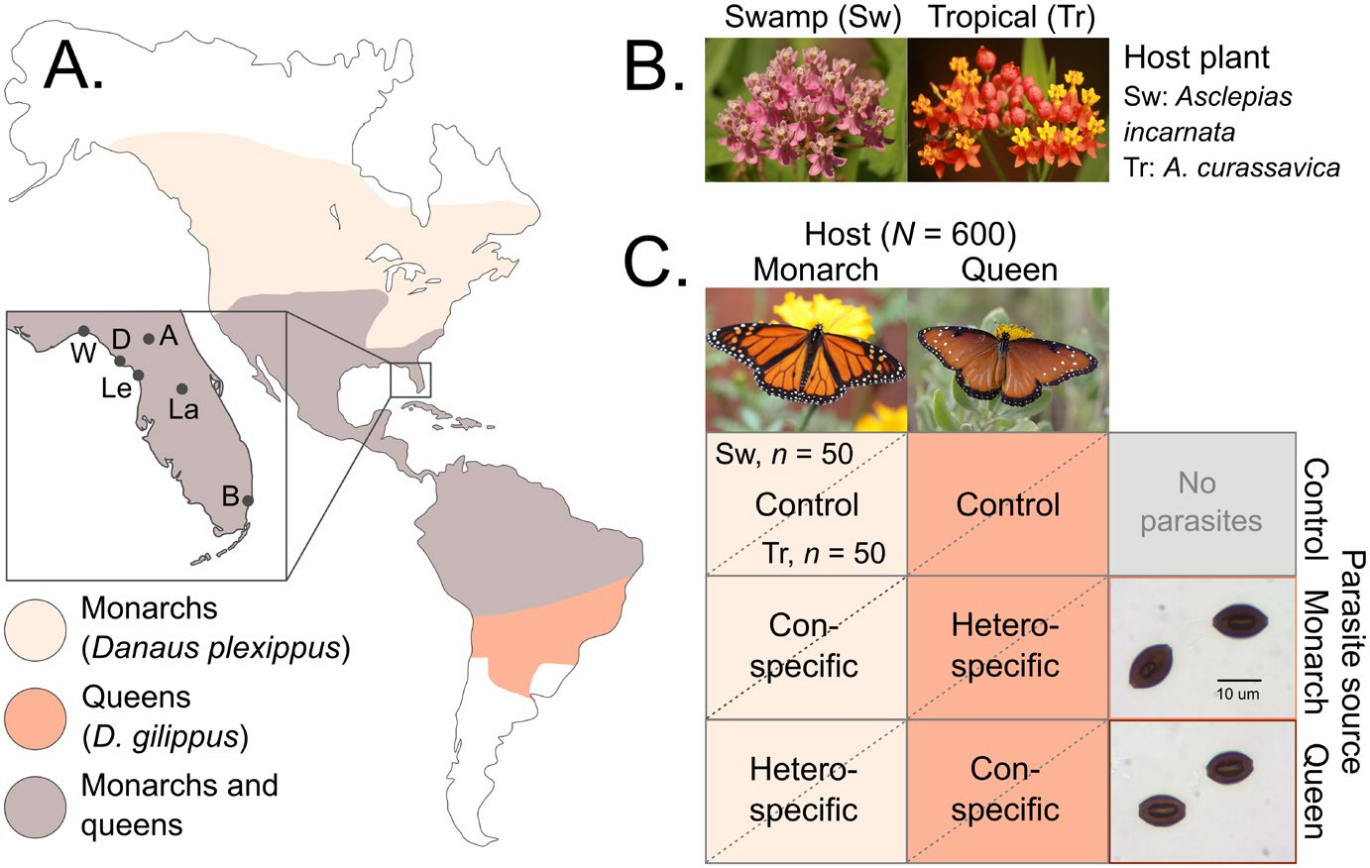
(A) The range of monarchs (light tan, *Danaus plexippus* ) and queens (orange, *D. gilippus* ) in the Americas. The overlapping range of monarchs and queens is shown in brown. Range maps were redrawn from (Jetz et al. 2012), and use data from (Balmer 2007; Brock and Kaufman 2006; Klimaitis et al. 2018). Inset shows the state of Florida and butterfly and parasite sources. *W* = Wakulla County, *A* = Alachua County, *B* = Broward County, *D* = Dixie County, *La* = Lake County, *Le* = Levy County. (B) Milkweed species used in this study; *Sw* = low‐cardenolide swamp milkweed ( *Asclepias incarnata* ) and *Tr* = high‐cardenolide tropical milkweed ( *A. curassavica* ). (C) Experimental design. Within each host species, larvae were chosen from either three (monarch) or twelve (queen) genetic lineages; for each parasite group, larvae were assigned to one of three isolates. Sample size was 50 larvae per treatment combination (*N* = 600 larvae). Butterfly and milkweed photographs by Icosahedron (2017), Flannery (2015), Mathias (2015), and Ramsey (2007). Images not to scale.

Neogregarine protozoan parasites (Neogregarinorida: Ophryocystidae) that infect milkweed butterflies (Lepidoptera: Nymphalidae: Danainae) are ideal for studying host specialization and cross‐species transmission. These host–parasite associations are geographically widespread (Altizer and de Roode 2015; Müller‐Theissen et al. 2025) and parasites show molecular divergence and partial specialization across host species (Mongue et al. 2023; Barriga et al. 2016). However, the degree of parasite differentiation across host species, realized host range, and extent of cross‐species transmission remain understudied. We focus on two congeneric butterfly species: monarchs ( *Danaus plexippus* ) and queens ( *D. gilippus* ). These species are ecologically similar (Brower 1961a; Brower 1962) and overlap geographically (Figure 1) (Jetz et al. 2012; Balmer 2007; Brock and Kaufman 2006; Klimaitis et al. 2018). Queens are not known to migrate long distances like migratory monarchs, but they can undertake regional, seasonal movements (Einem 2003; Hobson et al. 2021). The protozoan *Ophryocystis elektroscirrha* (*OE*) infects monarchs, with prevalence varying from 5%–30% in migratory populations and up to 75%–100% in resident (non‐migratory) populations (Altizer and de Roode 2015; Altizer et al. 2000). Parasites morphologically similar to *OE* (termed *OE*‐like) infect queens (Mclaughlin and Myers 1970), with reported prevalence of 20% (Barriga et al. 2016; Leong et al. 1997a) and up to 40% based on our field observations (unpublished). The molecular identity of *OE*‐like parasites infecting wild queens is unknown, but small‐scale experiments using monarchs and queens and their respective parasites suggested these parasites are at least partially specialized (Barriga et al. 2016; Leong et al. 1992).

*OE* and *OE*‐like parasites are transmitted when adult butterflies scatter spores (i.e., parasite oocysts) onto eggs and milkweed host plants, and the spores are then ingested by their offspring or unrelated larvae (either of the same or different species). Spores lyse in the gut, and parasites replicate internally. During the pupal stage, spores form around the scales of developing adult butterflies, and infected adults eclose covered with millions of spores on the outside of their bodies (Leong et al. 1997a; Leong et al. 1992). Fitness effects of *OE* in monarchs include smaller body sizes and reduced probability of mating (Altizer and Oberhauser 1999; Bradley and Altizer 2005; de Roode et al. 2007). *OE* has also been suggested as one cause of declines in the North American migratory monarch populations in recent decades (Majewska et al. 2019; Thogmartin et al. 2017; Brower et al. 2012; Schultz et al. 2017), due to increasing prevalence (Majewska et al. 2022) and reduced migration and survival in infected monarchs (Bradley and Altizer 2005; Altizer et al. 2015; Bartel et al. 2011). The high prevalence of *OE* in resident monarchs in the southern United States (US) creates exposure risks for migratory monarchs, primarily during spring recolonization (Satterfield et al. 2018). Similarly, high *OE* prevalence in monarchs could impact sympatric butterfly species like queens. Therefore, studies are needed to quantify the risk of cross‐species transmission of parasites and the fitness consequences of infection in different hosts.

In this study, we performed cross‐infection experiments between monarchs and queens using their respective parasites to measure the fitness consequences of cross‐species transmission and differential investment in immunity. We asked: (1) How do resistance and tolerance to infection differ between milkweed butterfly species exposed to parasites of conspecific versus heterospecific origin? (2) Does differential investment in immunity, as measured by melanization activity, hemocyte concentration, and differential cell type counts, mirror variation in infection between the two butterfy species? Considering the varying levels of cardenolide toxins in milkweed host plant species, we used both high and low cardenolide species to account for potential effects on host–parasite interactions [e.g., (de Roode et al. 2008a; Gowler et al. 2015)]. We predicted that queens would show greater resistance and higher investment in immunity than monarchs to both conspecific and heterospecific parasites, consistent with previous work (Barriga et al. 2016). We also predicted that trade‐offs between resistance and tolerance to infection could result in queens being less tolerant of infection than monarchs (Salgado‐Luarte et al. 2023; Raberg et al. 2007).

## 2 Methods

### 2.1 Host, Parasite, and Plant Sources

Butterflies used in this experiment were the offspring of wild monarchs and queens captured at six locations in Florida, US, during September 2020 (Figure 1). Queens in this region are sympatric with monarchs, sharing the same habitats either seasonally, with migrating monarchs, or year‐round, with resident monarchs (Brower 1961a; Brower 1962). These habitats primarily include pastures, roadsides, forest edges, and secondary growth.

Wild adults were collected using aerial insect nets. All butterflies were examined for infection by *OE*/*OE‐*like protozoans (Altizer et al. 2000), and only uninfected adults were used to obtain progeny. Adult females were returned to the laboratory already mated or set to mate in 0.6 m^3^ meshed cages in a naturally lit room and fed 20% honey solution *ad libitum*. Mated females were provided with greenhouse‐grown potted tropical milkweed ( *Asclepias curassavica* ) for oviposition to obtain eggs that represented full‐ and/or half‐sib genetic lineages. We obtained eggs from three female monarchs and 12 female queens. We had a lower number of founding monarchs owing to the high prevalence of *OE* in monarchs in the sampling area (80% in monarchs versus 23% in queens), resulting in only two parasite‐free females. Therefore, we supplemented the monarch numbers using eggs from a laboratory female (third generation derived from wild fall migrants captured in Florida in the previous year) mated to a healthy wild Florida male. Parasite spores were harvested directly from infected wild‐caught butterflies, also collected from the same locations in Florida (Figure 1). Larvae were inoculated with one of three parasite isolates obtained from either monarchs or queens (six isolates in total). Isolates and butterfly lineages were randomized across treatment combinations.

Larvae in Florida encounter both swamp milkweed ( *Asclepias incarnata* ) and tropical milkweed (Figure 1). These two milkweed species differ dramatically in their total concentration and diversity of cardenolides (Agrawal et al. 2021), which can affect the within‐host replication of *OE* (de Roode et al. 2008a; Hoogshagen et al. 2023) and monarch tolerance to infection (Sternberg et al. 2012). We therefore raised larvae to the adult stage using fresh cuttings of the low cardenolide swamp milkweed (cardenolide levels 0.007 μg/mg dry weight in latex) or the high cardenolide tropical milkweed (cardenolide levels 2.1 μg/mg) to examine how milkweed host plant species influenced the outcomes of exposure, immune defense metrics, and fitness metrics (Figure 1). Because differences in cardenolide concentration for these two milkweed species have been consistently established by prior work [e.g., (de Roode et al. 2008a; Züst et al. 2019)], we did not measure them directly here. Milkweed plants were raised from seeds, planted into 12.5 cm diameter pots, and used for the experiment when they were approximately 9 months old. Plants were greenhouse‐grown under approximately 28°C and 16‐h photoperiods under artificial lights and fertilized every 3 months with time‐released fertilizer (15–9‐12 NPK, 1 tablespoon per pot).

### 2.2 Experimental Design, Inoculation, and Rearing Protocol

Larvae remained on their natal milkweed host plant (tropical milkweed) until they reached the mid‐second instar stage (2–3 days post‐hatching). Larvae were then assigned to receive either no parasites, queen parasites, or monarch parasites. Within each host–parasite source combination, larvae were fed either swamp or tropical milkweed from the day of inoculation until pupation. This design resulted in 12 treatment combinations of butterfly species × parasite source × plant diet combinations (Figure 1). We randomized parasite and host lineages within each treatment and used 50 larvae per combination to initiate the experiment (*N* = 300 monarchs and 300 queens).

Second‐instar larvae were experimentally exposed to a controlled parasite dose (20 parasite spores per larva) (de Roode et al. 2007). Briefly, spores were manually transferred to 1 cm^2^ milkweed leaf pieces (swamp or tropical milkweed) inside 100 × 15 mm Petri dishes. Control larvae were fed swamp or tropical milkweed without spores. The larvae were transferred to individual dishes and remained until leaf material was consumed (≤ 48 h). Dishes were kept inside an environment chamber (Percival Intellus, Percival Scientific Inc., Perry, IA) set to 29.5°C day and 24°C night temperatures (13‐h days). Following inoculation, larvae were transferred to individual 0.47 L plastic containers with mesh screen lids and reared singly to the adult stage on fresh milkweed cuttings. The cuttings were soaked in a 20% bleach solution for 20 min and rinsed in tap water before use. Milkweed cuttings were changed every 1–2 days, and larvae were allowed to feed *ad libitum*. Containers were randomized across three environment chambers set to the same conditions previously described until adult eclosion. We recorded the pupation date, adult eclosion date, sex, and presence of wing deformity for adults that successfully eclosed from the pupal case.

### 2.3 Immune Assays

We removed and froze a subset of monarchs (*n =* 55) and queens (*n =* 51) at the second instar for future analysis (not described further in this study). Another subset of monarchs (*n* = 56) and queens (*n* = 53) from across treatment combinations was bled at the fifth (final) larval instar for immune assays and frozen afterward. Hemolymph was collected from larvae by clipping a tubercle at the base or puncturing a proleg with a 29 G 1/2″ needle. All larvae were weighed to the nearest 0.001 g to account for potential associations between body mass and immune defense metrics (we did not weigh larvae not assessed for immune metrics). Immunocompetence was assessed by counting immune cells (i.e., hemocytes) using a Kova hemacytometer chamber to estimate the number and cell type differentials of hemocytes per μL of hemolymph following published protocols (Satterfield et al. 2013; McKay et al. 2016a, McKay et al. 2016b). Insect hemocytes are mainly involved in phagocytosis and encapsulation of foreign invaders and participate in clot formation of external wounds (Lavine and Strand 2002). To estimate hemocyte concentration (cells/μL) and perform differential hemocyte counts (i.e., the proportion of each differentiated immune cell type; namely granulocytes, spheroid cells, plasmatocytes, and oenocytoids), 3 μL of hemolymph was diluted 1:10 in sterile Pringle's saline and loaded into 6.6 μL hemocytometer slides. The total number of hemocytes was counted under 400 × magnification on two separate chambers per sample, and the concentration was estimated as the average number of hemocytes per μL. Hemocyte types were distinguished by morphology, and the relative abundance of each cell type present was recorded.

We quantified the activity of the enzyme phenoloxidase (PO) as a separate metric of immune defense. In insects, melanization is catalyzed by PO and provides immune defense against bacteria, fungi, viruses, and larger parasites; it also plays a role in wound healing and pigmentation, among other biological functions (Cerenius and Soderhall 2004; Gonzalez‐Santoyo and Cordoba‐Aguilar 2012; Lu et al. 2014). For PO assays, 20 μL of hemolymph was diluted 1:1 in ice‐cold, sterile Pringles' saline (1× in 1 L dD H_2_0: 0.9 g NaCl, 0.2 g KCl, 0.2 g CaCl, 4.0 g dextrose) and stored at −80°C for up to 3 months. Samples were thawed on ice and rapidly processed by loading 10 μL of sample into 96‐well plates with 190 μL of buffer and an immune elicitor (in dD H20: 50 mM NaH_2_PO_4_ monobasic monohydrate adjusted to 6.5 pH, 2 mM dopamine, and *Micrococcus luteus* at 3% total volume). We measured the absorbance of the colorimetric reaction at 490 nm (A_490_) every 27 s at 30°C for 3 h (401 measures) using a microplate reader (EL808, BioTek Instruments Inc., Salt Lake City, UT). PO activity was quantified using the final average absorbance value and maximum slope of the kinetic curve (absorbance per read) during the linear phase of the melanization reaction (Satterfield et al. 2013; Siva‐Jothy 2000; Hall et al. 1995). The average maximum slope of the kinetic curve was obtained after fitting a Gompertz sigmoid function using non‐linear least squares and calculating the first derivative. PO assays were run twice per sample in separate runs, and the averages of the two assays per sample were used for the analysis.

### 2.4 Parasite Infection Metrics

We quantified infection in two ways to determine host resistance and parasite infectivity. First, butterflies were assigned a binary infection status based on the presence of spores (Altizer et al. 2000; de Roode et al. 2007). Starting 3 days before the expected eclosion date, pupae were checked daily for the presence of markings consistent with parasite infection and given a score of 0–5 according to predefined criteria (de Roode et al. 2009). The last (highest) score was used for further analysis. Upon eclosion, adults with a pupal score of 0, 1, or 2 were examined for the presence of infection by pressing a clear tape against the abdomen and counting the number of spores (oocysts) under a stereomicroscope, as described in (Altizer et al. 2000). Butterflies with a pupal score of 3–5, or with a tape sample showing 100 or more spores, were assigned to the infected class (de Roode et al. 2009; de Roode et al. 2008b). For all infected adults, we obtained a continuous measure of spore load using a shake‐and‐count method (de Roode et al. 2007) to estimate the total number of spores per infected butterfly.

### 2.5 Host Fitness Metrics

We measured adult forewing area (mm^2^), weight (g), lifespan (days), and the presence of wing deformity (0 = absent, 1 = present) as indicators of host fitness. These measures were used to inform analyses of host tolerance to infection. Tolerance was inferred by the slope of the association between spore loads and lifespan for the different host species in the subset of infected hosts. Adults were weighed to the nearest 0.001 g one day post‐eclosion to allow for the release of the meconium. Adults were placed into individual glassine envelopes after their wings hardened, kept at 13°C without food, and checked daily to record their lifespan in days. This measure of lifespan reflects both the duration of adult life and the starvation resistance derived from stored larval energy reserves. Prior work showed this measure is similar to the effect of *OE* infection on monarch lifespan when butterflies are held in outdoor flight cages and fed *ad libitum* (de Roode et al. 2009).

### 2.6 Statistical Analysis

We first examined how design variables (host, parasite, and milkweed species) predicted pre‐adult mortality and infection status, and how they predicted spore loads for the subset of infected butterflies. Next, we examined immune defense metrics in larvae, followed by adult fitness metrics. Last, we tested for differences in tolerance in infected adult butterflies. For all response variables, we tested for normality using the Shapiro–Wilk normality test and calculated appropriate summary statistics [median ± interquartile range, mean ± standard error, or proportions]. We also performed bivariate analyses (Wilcoxon rank sum test or Pearson's Chi‐squared test) to examine how different response variables differed across design levels and examined the correlation between variables using the Spearman's rank correlation coefficient (*r*_*s*_).

We fitted general and generalized linear models (GLMs: logistic, Poisson, quasi‐Poisson, or negative binomial, as appropriate, to account for different error distributions) to examine how different response variables differed across design levels. Our full model included host species, parasite (exposure or infection) source, milkweed species (larval diet), (immature or adult) weight, and sex (adults only) as predictors. This model also included linear interaction terms across the three design variables, mainly host species × parasite source, host species × milkweed species, and parasite source × milkweed species. Since infection can only be determined in adults, we used parasite exposure source (levels: control, monarch parasites, or queen parasites) as a predictor for larval measures and exposure outcomes. For adult measures, parasite infection outcome (levels: control/uninfected, monarch parasites, or queen parasites) was used. We did not fit models with interaction terms when analyzing the presence of wing deformity owing to the low number of observations. The models did not include host and parasite lineages as random effects because treatments were randomized across host and parasite genotypes.

We added immature weight as a covariate for analyzing larval immune defense metrics and adult weight for analyzing adult immune and fitness metrics. Both variables were centered and scaled. The sex of butterflies was added as a covariate in analyses of adult measures. To analyze tolerance to infection, we added the interaction terms for host species × spore load, milkweed species × spore load, and parasite source × spore load.

For all likelihood‐based models, we performed model selection using the corrected Akaike information criterion (AICc) value to choose between a set of hypothesized fixed‐effects models with or without the inclusion of two‐way interaction terms of the three design variables (i.e., combinations of host species, parasite, and milkweed host plant species treatment). For quasi‐likelihood models, we looked for significant differences among candidate models using an *F*‐test (ANOVA) (Crawley 2013) and kept the simplest model. The goodness of fit of models was assessed using the variance explained: coefficient of determination (*R* ^*2*^) for linear models, Tjur's *R* ^*2*^ for logistic models, and Nagelkerke *R* ^*2*^ for models with a negative binomial distribution. We also assessed the goodness of fit of linear models using diagnostic plots, performed Hosmer and Lemeshow tests for logistic regression models, and examined the residual deviance in Poisson, negative binomial, and quasi‐Poisson models. Statistical analyses were performed using R version 4.0.4, base R (R Core Team 2024), and packages tidyverse version 2.0.0 (Wickham et al. 2019), ResourceSelection version 0.3–6 (Lele et al. 2019), sjPlot version 2.8.15 (Lüdecke 2021), AICcmodavg version 2.3–3 (Mazerolle 2020), corrr version 0.4.4 (Kuhn et al. 2022), and MASS version 7.3–60 (Venables and Ripley 2002). Data were visualized using packages ggplot2 version 3.4.4 (Wickham 2016) and patchwork version 1.2.0 (Pedersen 2024).

## 3 Results

**FIGURE 2:**
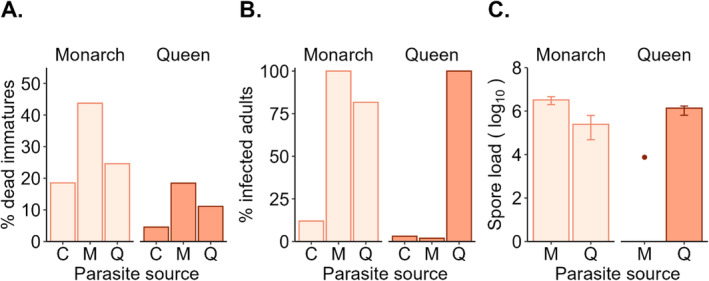
The outcome of exposure to conspecific and heterospecific parasites by source and host species. (A) Pre‐adult mortality (larva or pupae stage). (B.) Percentage of infected adults. (C) Average spore load (log_10_) in infected adults. Error bars show interquartile ranges. *C* = control, *M* = monarch parasites, *Q* = queen parasites.

Of the 385 larvae (*n =* 189 monarchs and 196 queens) not subjected to immune assays, 29% of monarchs and 11% of queens died before eclosion (larva or pupa stage; *χ* ^*2*^ (1, 76) = 17.196, *p* < 0.01). Monarchs had a significantly greater probability [odds ratio (OR) = 3.41] of pre‐adult mortality compared to queens. Larvae exposed to monarch parasites (OR = 3.89), but not those exposed to queen parasites, had a significantly greater probability of dying. Hosts that fed on tropical milkweed also had a significantly greater probability (OR = 1.95) of pre‐adult mortality than those that fed on swamp milkweed (Figure 2 and Table A1). Of the 71% of monarchs and 89% of queens that survived to adulthood, 12% of monarchs and 3% of queens in the control group were infected and excluded from further analysis (*n* = 8/115), while 89% of monarchs and 52% of queens in the parasite‐treated groups were infected by either conspecific or heterospecific parasites (*n* = 133/194).

### 3.1 Infection Outcomes

All monarch larvae exposed to conspecific parasites (*n* = 36) and 82% (*n* = 40/49) of monarchs exposed to heterospecific parasites [*χ* ^*2*^ (1, 90) = 6.125, *p* = 0.013] eclosed as infected adults. All queen larvae exposed to conspecific parasites (*n* = 56) but only 2% (*n* = 1/52) exposed to heterospecific parasites [*χ* ^*2*^ (1, 110) = 102.16, *p* < 0.001] eclosed as infected adults (Figure 2). Consistent with the observations, the model indicated that monarchs had a significantly higher probability of infection than queens (IRR = 50.17), especially in response to heterospecific parasites (significant interaction; Table A1). Queens exposed to queen parasites had a higher probability of infection compared with queens exposed to monarch parasites (IRR = 46.69) (Table A1). Milkweed species (larval diet), sex, and adult weight did not predict infection probability (Table A1).

In infected hosts, spore loads were positively correlated with pupal scores (*r*_*s*_ = 0.62). Spore loads were higher in hosts infected by conspecific versus heterospecific parasites, with a significant interaction between host and parasite source (Figure 2 and Table A1). Monarchs had significantly higher spore loads than queens (Table A1), with the highest spore loads observed in monarchs infected by monarch parasites (Figure 2). Only one queen was infected by monarch parasites, limiting our ability to compare spore loads between the two parasite types in queens. Milkweed species and adult weight did not predict differences in spore load (Table A1).

### 3.2 Immune Defense of Larvae Exposed to Parasites

**FIGURE 3:**
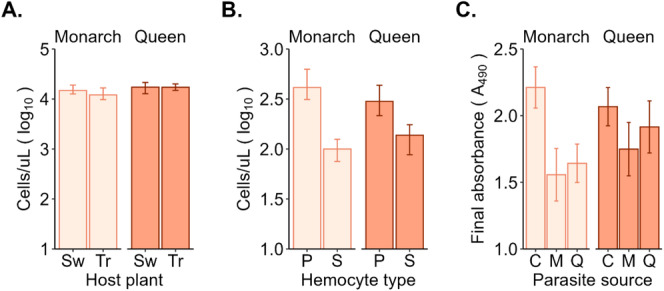
Immune defense metrics of fifth‐instar larvae by host species. (A) Median concentration of hemocytes (cells/μL) by milkweed species (larval diet). (B) Median plasmatocyte and spheroid cell concentration. (C) Average final absorbance (A_490_) of the melanization reaction by parasite source. Error bars show interquartile ranges for (A) and (B) and standard errors for (C). Milkweed species: Sw = low‐cardenolide swamp milkweed, *Tr* = high‐cardenolide tropical milkweed. Hemocyte type: *P* = plasmatocytes, *S* = spheroid cells. Parasite source: *C* = control, *M* = monarch parasites, *Q* = queen parasites.

Hemocyte concentration was positively correlated with PO final absorbance (*r*_*s*_ = 0.4) and the maximum slope of the melanization reaction (*r*_*s*_ = 0.4; Table A3). Monarchs had lower hemocyte concentration (10^4.15^ cells/μL) than queens (10^4.24^ cells/μL; Wilcoxon rank sum test, *W* = 2022.5, *p* = 0.002), but this difference was not significant after adjusting for covariates (Table A1). Parasite source, milkweed species, and larval weight did not predict hemocyte concentration differences (Figure 3), but monarchs that fed on tropical milkweed had significantly lower hemocyte concentration (IRR = 0.75) than those that fed on swamp milkweed.

Granulocytes were the most abundant hemocyte type (66%), followed by plasmatocytes (24%), spheroid cells (7%), and oenocytoids (3%). Hemocyte differentials did not differ between host species across cell types [granulocytes (*χ* ^*2*^ (54, 110) = 59.555, *p* = 0.281); plasmatocytes (*χ* ^*2*^ (51, 110) = 54.048, *p* = 0.359); spheroid cells (*χ* ^*2*^ (21, 110) = 27.203, *p* = 0.164); oenocytoids (*χ* ^*2*^ (12, 110) = 11.863, *p* = 0.457)]. The concentrations of plasmatocytes and spheroid cells were negatively correlated (*r*_*s*_ = −0.26; Table A3). Granulocytes were positively correlated with the final absorbance value of the melanization reaction (*r*_*s*_ = 0.32).

Monarch larvae had significantly higher densities of plasmatocytes (IRR = 1.55) but lower densities of spheroid cells (IRR = 0.75) than queens (Figure 3; Table A1). Granulocyte and oenocytoid densities did not differ between host species. Heavier larvae had greater granulocyte densities (IRR = 1.08). Larvae exposed to monarch parasites (IRR = 1.39) had significantly higher spheroid cell densities, but those that fed on tropical milkweed had lower spheroid cell concentration (significant interaction). Oenocytoid concentration was not associated with any of the variables tested.

**FIGURE A1:**
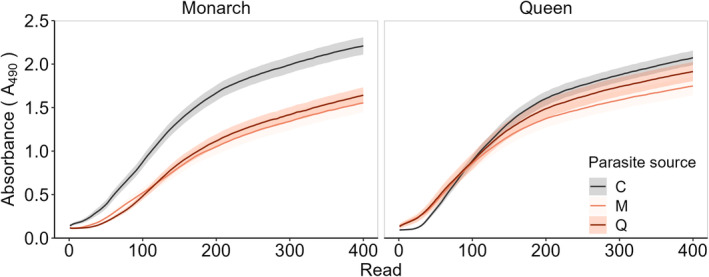
Kinetic curve of the melanization reaction (average absorbance per read ± standard errors) by host species and parasite source. The final absorbance value and the maximum slope of the melanization kinetic curve were highly correlated (r_s_ = 0.9). Parasite source: C = control, M = monarch parasites, Q = queen parasites.

Given the strong positive correlation between the final absorbance value and maximum slope of the melanization kinetic curve (*r*_*s*_ = 0.9; Table A3 and Figure A1), the final absorbance was used as the measure of PO activity. Monarch larvae had significantly lower final absorbance values than queens, and larvae exposed to parasites had lower absorbance than unexposed larvae (Figure 3 and Table A1). PO absorbance increased with larval weight, but milkweed species did not predict differences in melanization.

### 3.3 Butterfly Fitness and Tolerance to Infection

**FIGURE A2:**
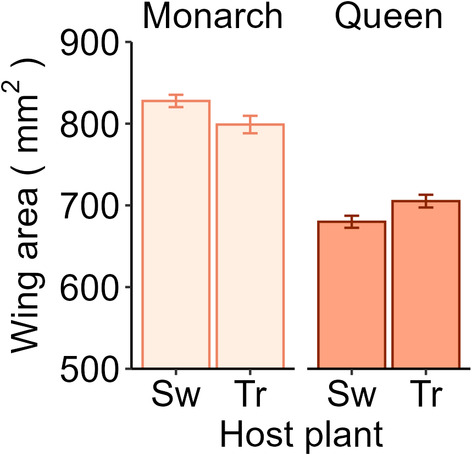
Mean wing area by host species and milkweed host plant. Error bars show standard errors. Milkweed host plant species: Sw = low‐cardenolide swamp milkweed, Tr = high‐cardenolide tropical milkweed.

Wing area was positively correlated with adult weight (*r*_*s*_ = 0.9) and wing length (*r*_*s*_ = 0.93; Table A2). Monarch adults were significantly larger than queens, with a mean wing area of 815.5 mm^2^ in monarchs and 691.9 mm^2^ in queens [*F* (1, 295) = 216.2, *p* < 0.001]. Parasite source did not predict differences in adult forewing area. Adult queens that fed on tropical milkweed as larvae were significantly larger than those that fed on swamp milkweed, but the reverse was true for monarchs (significant interaction; Table A1 and Figure A2). On average, males were significantly larger than females.

**FIGURE 4:**
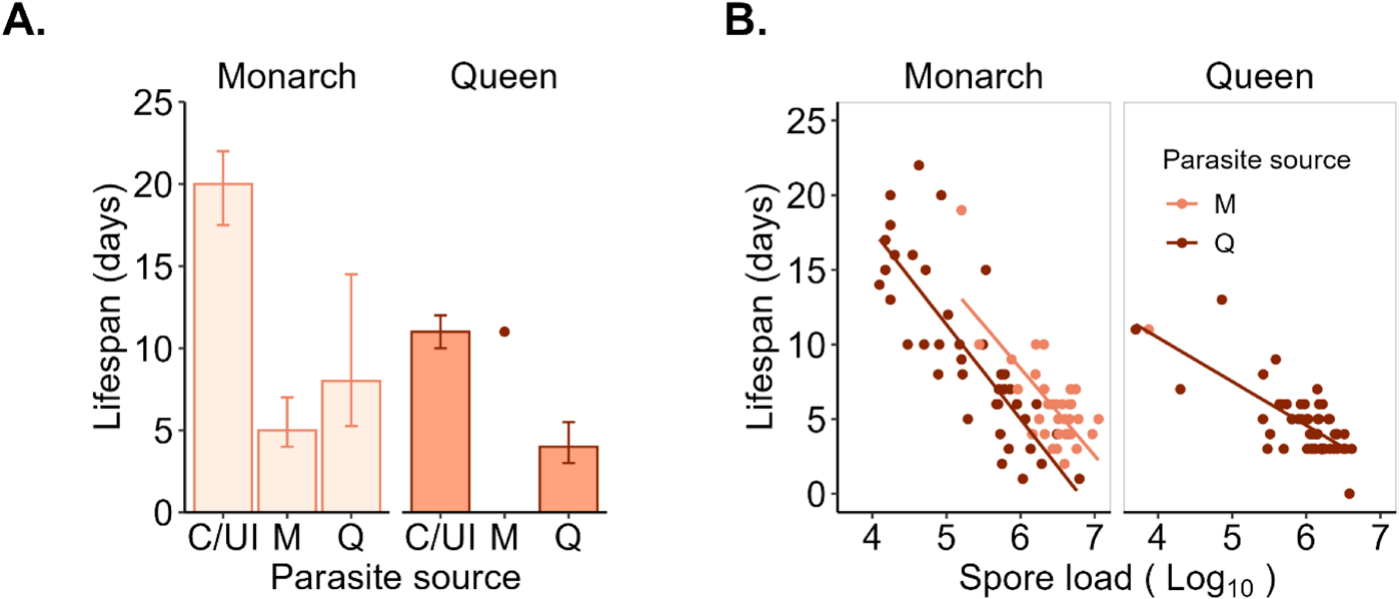
Fitness and tolerance of adult butterflies by host species and parasite source. (A) Median adult lifespan (days). Error bars show interquartile ranges. (B) Tolerance, as measured by the relationship between spore loads and lifespan. Parasite source: C/U = control/uninfected, *M* = monarch parasites, *Q* = queen parasites.

Adult lifespan was positively correlated with adult weight at eclosion (*r*_*s*_ = 0.37; Table A3). The median lifespan was 14 days for monarchs and 10 days for queens (*W* = 8475.5, *p* < 0.01). Monarchs had significantly longer lifespans than queens (IRR = 1.28; Figure 4 and Table A1). Adults infected by queen parasites had significantly shorter lifespans than uninfected adults (IRR = 0.43). Monarchs infected by conspecific parasites had significantly shorter lifespans than uninfected monarchs (IRR = 0.36; significant interaction). Monarchs experienced a 75% median lifespan reduction when infected by conspecific parasites compared with 60% with heterospecific parasites. Queens experienced a 64% median lifespan reduction when infected by conspecific parasites (only one queen became infected by heterospecific parasites). Larval diet and sex did not predict lifespan differences.

Among infected adults, lifespan was negatively correlated with spore load (*r*_*s*_ = −0.50). Infected monarchs (IRR = 0.78) and hosts of either species infected by queen parasites (IRR = 0.73) had significantly lower lifespans (Table A1). The negative slope of the relationship was steeper for monarchs relative to queens (IRR = 0.8; Figure 4), indicating that monarchs were less tolerant of parasite infection. The relationship between spore load and lifespan did not depend on parasite source or milkweed species.

Nine percent of monarchs and 4% of queens eclosed with wing deformity (*χ* ^*2*^ (1, 315) = 2.8197, *p* = 0.093). Hosts infected by monarch parasites had a greater probability (IRR = 8.51) of eclosing with wing deformity (Table A1). Milkweed species, adult weight, and sex did not predict the probability of emerging with wing deformity. Among infected adults, those with higher spore load, irrespective of host or parasite source, had a significantly greater probability (IRR = 13.59) of emerging with wing deformity (Table A1).

## 4 Discussion

Our study showed that host and parasite identity influence resistance, tolerance, specificity, and virulence in this host–parasite interaction, building on recent work in this area (Antonovics et al. 2013; Lambrechts et al. 2006; Little et al. 2010). We found evidence for imperfect and asymmetrical parasite specialization, with parasites from both host species infecting and replicating better within their conspecific hosts. These results have implications for the conservation and infection dynamics of monarchs and queens at both the population and community levels. Notably, only queen parasites were able to consistently infect both host species, underscoring the high specificity of monarch parasites for their natal hosts, as well as the heightened susceptibility of monarchs to both conspecific and heterospecific parasites. While there was one instance of monarch parasites infecting queens, we believe this case is inconclusive due to the presence of infections in the controls.

Monarchs suffered substantial fitness loss from infection, particularly with their own parasites, consistent with previous studies linking *OE* replication to reduced monarch lifespan (Altizer and Oberhauser 1999; de Roode et al. 2007; de Roode et al. 2009). Queens showed greater tolerance to infection, suggesting they can survive well enough to effectively transmit parasites within and between host species. Given the increasing prevalence of *OE* in North American monarchs and its negative impact on migration (Majewska et al. 2022), along with the overlap in range, breeding sites, and host plants between monarchs and queens, this study provides a necessary basis for predicting the risks of cross‐species transmission. Our results align with previous findings that queens are moderately resistant to conspecific parasites but highly resistant to heterospecific ones (Barriga et al. 2016). The low likelihood of monarch parasites to infect queens could arise early in the infection process due to differences in gut pH or permissiveness of the gut lining to sporozoite penetration. Alternatively, it could be due to the failure of parasites to complete asexual cycles of within‐host replication or sexual cycles in the hemolymph or hypoderm in queens (Smith and Cook 2008; Clopton and Gold 1996; Steele et al. 2012; Clopton and Gold 1995; Patil et al. 1985). Importantly, the strong resistance of queens to monarch parasites observed in this study goes beyond that reported from prior genotype‐specific interactions. In particular, our findings contrast with those from Mclaughlin and Myers (1970), who reported high infection rates in both monarchs and queens at Archbold Biological Station in Florida and no indication of host specificity.

Queens showed stronger innate immune defense than monarchs, which could explain their overall greater resistance to infection but not their differential response to conspecific versus heterospecific parasites. Queen larvae showed a stronger melanization response and higher spheroid cell concentration than monarchs, suggesting different immune defense strategies between the two species. Whereas granulocytes are phagocytic cells and plasmatocytes engage in the aggregation and encapsulation of foreign invaders (Lavine and Strand 2002), the precise role of spheroid cells in insect immunity remains unclear. Queens also exhibited a non‐significant trend toward higher hemocyte concentration than monarchs. In monarch larvae, higher hemocyte concentrations have been linked to lower spore loads in adults (Altizer and de Roode 2015; McKay et al. 2016a), indicating a role of hemocytes in the response against replication of *OE* and, presumably, *OE*‐like parasites. Similarly, in mosquitoes infected with *Plasmodium*, another Apicomplexan protozoan, melanization of sporozoites in the midgut can prevent further infection (Dos Santos et al. 2024; Simões et al. 2017). Thus, the elevated innate immune defenses observed in queens could explain why they exhibited lower infection probability and spore loads.

Conversely, the efficacy of parasites in evading the larval immune system could explain their success. In monarch‐*OE* interactions, older larvae show higher resistance to infection (Leong et al. 1997b) and increased hemocyte concentration and PO activity (Altizer and de Roode 2015). These findings suggest that some parasites may evade or overcome defenses in younger instars, whereas parasite invasion and replication are effectively controlled by older instars. One limitation of our study is that we only measured immune responses in fifth‐instar larvae and assumed that general innate immune measures predict responses to *OE* and *OE*‐like parasites (Mallon et al. 2003; Schmid‐Hempel and Ebert 2003). Future studies focusing on the host's immune defense and other processes early in the infection (e.g., in first‐ and second‐instar larvae) could provide greater insight into the mechanisms of infection.

Our finding that queens were both more tolerant and more resistant to parasites than monarchs was unexpected. Instead, we predicted that queens would be more resistant to infection but that monarchs would better tolerate *OE* infection, given possible trade‐offs between resistance and tolerance [e.g., (Salgado‐Luarte et al. 2023; Raberg et al. 2007)]. For example, past work on monarchs showed a negative relationship between lipid reserves and PO activity in fall migrating adults, suggesting that monarchs are limited energetically to invest in either parasite defense or fat storage (which could increase tolerance to infection by prolonging lifespan), but not both (Satterfield et al. 2013). Other work (McKay et al. 2016a, McKay et al. 2016b) showed that monarch immune defenses are costly for flight, development, and survival when food resources are limited. For these reasons, we expected that butterflies that invest in greater immune defense might have a reduced ability to tolerate infection. However, experimental findings for queens counter this hypothesis.

Although the mechanisms underlying differences in tolerance between monarchs and queens are unclear, it is possible that resistance and immune defense are more costly in monarchs than queens. Queens are smaller bodied and have shorter lifespans in the absence of infection. It might be that monarchs trade off larger size and greater longevity, both of which are important in successful migration (Li et al. 2016; Altizer and Davis 2010; Herman and Tatar 2001), against other fitness components such as resistance and tolerance to infection. This trade‐off may remain relevant in resident populations, as migratory traits (including larger wing sizes) are genetically conserved even when environmental conditions do not trigger their developmental expression (Freedman et al. 2017). Similar trade‐offs have been observed in other systems. For example, shorter lifespans have been observed in yellow dung flies ( *Scathophaga stercoraria* ) artificially selected for increased PO activity (Schwarzenbach and Ward 2006). Wild populations of brown‐headed cowbirds ( *Molothrus ater* ) exhibited heightened resistance to several arboviruses compared to other closely related bird species but had shorter lifespans (Hahn and Smith 2011; Reisen and Hahn 2007). In Mediterranean field crickets (*Gryllus bimaculatus*) (Rantala and Roff 2005), body sizes were negatively correlated with encapsulation rates but positively correlated with lytic enzyme activity, also suggesting potential trade‐offs between immune functions. Further investigations are warranted to explore the consistency of our observations regarding resistance and tolerance in monarchs and queens from other sympatric populations (e.g., in Central and South America) and their implications for infection dynamics.

Although the genetic background of our founding monarchs was likely mixed, reflecting the influence of both migratory and resident lineages, variation in host–parasite genetic interactions across geographical regions could also account for differences in host susceptibility to infection between monarchs and queens. Specifically, queens might benefit from greater opportunities for adaptation to locally abundant parasite strains due to their more limited dispersal [but see (Pfeiler 2024)] and potentially higher site fidelity throughout their annual cycle. In contrast, migratory monarchs breed across a vast range in North America, show some population differentiation during the summer breeding period, but experience high genetic mixing during migration and overwintering [e.g., (Eanes and Koehn 1978)]. Importantly, past work showed evidence for genetic heterogeneity in both host resistance and parasite virulence in North American monarchs, producing strong genotypic interactions between monarch hosts and parasites (de Roode and Altizer 2010; Sternberg et al. 2013). This work showed that monarchs could exhibit resistance to some, but not all, parasite strains, suggesting an underlying genetic constraint on directional selection for resistance in this species. Resident monarch populations show genetic differentiation from migratory populations (Zhan et al. 2014) and may have greater opportunities for local adaptation. However, this potential may be counterbalanced by periodic influxes of migrant genes during fall, when resident monarchs encounter and breed with incoming individuals from migratory populations (Knight and Brower 2009; Zanden et al. 2018), potentially homogenizing genetic differences and limiting local adaptation to parasites.

Milkweed butterflies have a close relationship with their host plants, which can influence resistance and tolerance to infection (de Roode et al. 2008a; Hoogshagen et al. 2023; Sternberg et al. 2012). Previous studies showed that milkweeds with high diversity and concentration of cardenolides (especially tropical milkweed) can suppress parasite replication and improve host fitness (de Roode et al. 2008a; Hoogshagen et al. 2023; Sternberg et al. 2012). Surprisingly, we found no effect of milkweed species on parasite replication or host tolerance to infection. The relatively high exposure dose used may have overwhelmed the effects of host plant chemistry on parasite replication. Moreover, larvae were not placed on their assigned host plant species until the inoculation step, possibly obscuring plant‐specific effects (de Roode et al. 2011). Alternatively, parasites from different host species could be adapted to cardenolide profiles from different milkweed species across geographic locations (de Roode et al. 2008a; de Roode and Altizer 2010; Sternberg et al. 2013). In a similar fashion, monarch populations can show increased survival and development rates on commonly available host plants across their global range compared to plants they encounter less often (Freedman et al. 2020). Importantly, parasites collected in this study were all from sites in Florida that might have similar host plant assemblages (Cohen 1985). Further research is needed to test the possibility of parasite local adaptation to abundant host plant species from different geographic regions.

Our finding that tropical milkweed negatively affected hosts suggests a cost of storing or converting plant‐derived compounds (Agrawal et al. 2021; Tao et al. 2016; Zalucki and Brower 1992). Monarchs experienced more adverse effects of a diet of tropical milkweed compared with queens, possibly due to queens' lower cardenolide sequestration, different selectivity of cardenolides sequestered, and adaptations to use other plant‐derived defense compounds, such as pyrrolizidine alkaloids (Petschenka and Agrawal 2015; Petschenka et al. 2013; Mebs et al. 2012; Lawson et al. 2021; Boppré et al. 2022). Beyond fitness impacts, further research is needed to explore the implications of these interspecific differences on resistance and tolerance to parasites, including investment in immunity. For example, transcriptomic analysis suggests that, even though there are no differences in immune expression between *OE* infected and uninfected monarchs, they face a trade‐off between cardenolide detoxification and immunity, particularly when eating a high cardenolide diet. This trade‐off results in the downregulation of a small number of genes associated with immunity, while detoxification genes are upregulated (Tan et al. 2019). It remains uncertain whether queens face similar trade‐offs.

Overall, our findings indicate that monarch parasites were highly specific and, even following direct exposure to a relatively high dose of spores, unlikely to infect queens. This could be partly due to queens being better defended immunologically or biochemical and molecular incompatibilities between hosts and parasites. Monarchs and queens overlap in their geographic ranges across several regions, including the southern US (Florida, Texas, and Arizona), California, Central America, and northern South America. In these areas, the two species often share habitats and milkweed host plants for reproduction, creating numerous opportunities for cross‐species exposure to parasites from either host (Brower 1961a; Brower 1962; Cohen 1985; Brym et al. 2020; Young 1974). Since spores can remain viable on milkweed for months (Sanchez et al. 2021; Satterfield et al. 2017), exposure to heterospecific parasites does not require temporal overlap. This is relevant because early studies (Brower 1961a; Brower 1962) in populations of monarchs and queens in south‐central Florida suggested that temporal and spatial segregation could partially alleviate direct competition for resources between the two species. This pattern wasevident by an increase in queen populations when monarch populations decreased, along with shifts in milkweed preferences associated with changes in the relative abundance of each butterfly species. Additionally, these studies identified intra‐ and interspecific egg predation in larvae of both species (more frequent in queens) (Brower 1961b) as a potential density‐dependent regulating factor. Further research is needed to clarify the outcomes of both direct and apparent (i.e., parasite‐mediated) competition between these species, accounting for differences in life‐history traits such as longevity and fecundity.

*OE* has been extensively studied in monarchs since the 1990s, and the monarch‐protozoan interaction has become a model system for studying the effects of animal migration and host plant chemistry on animal‐pathogen interactions [e.g., (Altizer et al. 2000; de Roode et al. 2007; Altizer et al. 2015; de Roode et al. 2009)]. Even though the initial description of this parasite was based on infections in wild monarchs and queens in southern Florida in 1966 (Mclaughlin and Myers 1970), scientific understanding of *OE*‐like parasites infecting queens and other milkweed butterflies (Müller‐Theissen et al. 2025; Mongue et al. 2023; Barriga et al. 2016; Ndatimana et al. 2022), and the potential for cross‐species infections (Barriga et al. 2016; Gao et al. 2020) in natural populations remains limited. Our results contribute to understanding how closely related parasites can differ in their infectivity to the same or closely related hosts. These differences have implications for host and parasite fitness, as well as host range, consistent with findings in other systems, such as microsporidians infecting shrimp (Lievens et al. 2018) and fungal parasites infecting plants (Bruns et al. 2021). Future research should focus on identifying when infection fails—for example, during the initial invasion of the gut or later in the replication cycle—and understanding the mechanisms of parasite clearance and tolerance across host species. Research should also explore parasite genetic divergence, cross‐species exposures, and transmission dynamics in natural populations. Mathematical modeling can further elucidate the contributions of monarchs and queens to parasite dynamics within and between host species, informing conservation strategies for these butterfly species.

## 5 Author Contributions

**Maria L. Müller‐Theissen:** conceptualization (equal), funding acquisition (supporting), data curation (lead), formal analysis (lead), investigation (lead), methodology (equal), project administration (lead), resources (supporting), software (lead), validation (lead), visualization (lead), writing – original draft (lead), writing – review and editing (lead). **Nicole L. Gottdenker:** funding acquisition (supporting), supervision (supporting), writing – review and editing (supporting). **Sonia M. Altizer:** conceptualization (equal), funding acquisition (lead), methodology (equal), resources (lead), supervision (lead), writing – review and editing (lead).

## Conflicts of Interest

The authors declare no conflicts of interest.

## Data Availability

Data and code used in this manuscript are available from the Dryad Repository https://doi.org/10.5061/dryad.vhhmgqp2t

## Appendix A

**TABLE A1:** Fixed effects model results for infection outcomes (yellow), immune defense metrics (green), and fitness‐related metrics (purple). Significant associations are shown in bold.

Response	Model family	Predictors	Estimate	Standard error	95% confidence interval	*p*	*N*	R^2^	AICc	Delta AICc
			**OR**							
Pre‐adult mortality (0/1)	Binomial (link = logit)	Intercept	0.04	0.02	0.02–0.09	**< 0.001**	377	0.114	354.522	7.24
		Host [monarch]	3.41	0.98	1.97–6.10	**< 0.001**				
		Parasite [M]	3.89	1.41	1.96–8.16	**< 0.001**				
		Parasite [Q]	1.76	0.67	0.84–3.81	0.142				
		Milkweed [Tr]	1.95	0.54	1.14–3.40	**0.016**				
			**IRR**							
Outcome of exposure (0/1)	Quasipoisson (link = log)	Intercept	0.02	0.01	0.01–0.05	**< 0.001**	178	0.829	NA	NA
		Host [monarch]	50.17	29.57	18.11–196.13	**< 0.001**				
		Parasite [Q]	46.69	26.37	17.98–176.57	**< 0.001**				
		Milkweed [Tr]	0.75	0.23	0.41–1.36	0.345				
		Sex [male]	1.08	0.12	0.87–1.34	0.509				
		Adult weight	0.97	0.09	0.81–1.16	0.700				
		Host [monarch] × parasite [Q]	0.02	0.01	0.004–0.04	**< 0.001**				
		Parasite [Q] × Milkweed [Tr]	1.28	0.35	0.75–2.20	0.359				
		Host [monarch] × Milkweed [Tr]	1.23	0.29	0.78–1.96	0.369				
			**Coeff**.							
Log_10_ spore load (cells) —subset of infected	Linear	Intercept	3.86	0.57	2.73–4.99	**< 0.001**	120	0.394	209.77	26.43
		Host [monarch]	2.55	0.59	1.38–3.72	**< 0.001**				
		Parasite [Q]	2.02	0.57	0.90–3.14	**0.001**				
		Milkweed [Tr]	0.10	0.10	−0.10 – 0.31	0.314				
		Sex [male]	−0.01	0.11	−0.24—0.21	0.911				
		Adult weight	−0.06	0.09	−0.25 – 0.12	0.506				
		Host [monarch] × parasite [Q]	−3.09	0.58	−4.24 – 1.94	**< 0.001**				
			**IRR**							
Hemocytes concentration (cells/μL)	Negative binomial (link = log)	Intercept	18919.92	1998.72	15481.09–23246.63	**< 0.001**	109	0.222	2200.74	8.93
		Host [monarch]	0.97	0.11	0.76–1.24	0.813				
		Parasite [M]	0.97	0.08	0.82–1.14	0.698				
		Parasite [Q]	1.00	0.08	0.85–1.17	0.985				
		Milkweed [Tr]	1.06	0.10	0.87–1.27	0.570				
		Larval weight	0.91	0.09	0.74–1.14	0.388				
		Host [monarch] × Milkweed [Tr]	0.75	0.10	0.58–0.98	**0.032**				
			**IRR**							
Granulocytes concentration (cells/μL)	Negative binomial (link = log)	Intercept	1117.70	50.87	1021.25–1224.45	**< 0.001**	109	0.145	1490.81	8.38
		Host [monarch]	0.92	0.05	0.83–1.02	0.099				
		Parasite [M]	1.00	0.05	0.92–1.10	0.969				
		Parasite [Q]	1.02	0.05	0.93–1.11	0.738				
		Milkweed [Tr]	1.05	0.04	0.98–1.13	0.178				
		Larval weight	1.08	0.03	1.03–1.14	**0.003**				
			**IRR**							
Plasmatocytes concentration (cells/μL)	Negative binomial (link = log)	Intercept	331.97	37.47	265.54–417.46	**< 0.001**	109	0.187	1448.13	8.46
		Host [monarch]	1.55	0.20	1.18–2.04	**0.001**				
		Parasite [M]	0.90	0.10	0.72–1.12	0.352				
		Parasite [Q]	0.97	0.11	0.78–1.21	0.764				
		Milkweed [Tr]	0.97	0.09	0.81–1.16	0.742				
		Larval weight	0.93	0.06	0.81–1.07	0.267				
			**IRR**							
Spheroids concentration (cells/μL)	Negative binomial (link = log)	Intercept	126.05	15.75	99.15–162.01	**< 0.001**	109	0.240	1196.01	2.93
		Host [monarch]	0.75	0.09	0.59–0.96	**0.021**				
		Parasite [M]	1.39	0.22	1.02–1.88	**0.034**				
		Parasite [Q]	1.09	0.17	0.80–1.47	0.598				
		Milkweed [Tr]	1.30	0.20	0.96–1.75	0.087				
		imm Weight	0.99	0.06	0.88–1.12	0.891				
		Parasite [M] × Milkweed [Tr]	0.61	0.13	0.40–0.94	**0.024**				
		Parasite [Q] × Milkweed [Tr]	0.72	0.16	0.47–1.10	0.129				
			**IRR**							
Oenocytoid concentration (cells/μL)	Negative binomial (link = log)	Intercept	56.19	7.18	43.61–72.92	< 0.001	109	0.055	1055.96	7.82
		Host [monarch]	1.05	0.15	0.79–1.40	0.749				
		Parasite [M]	0.86	0.11	0.67–1.11	0.256				
		Parasite [Q]	1.05	0.13	0.82–1.35	0.710				
		Milkweed [Tr]	1.07	0.11	0.87–1.32	0.506				
		Larval weight	0.92	0.07	0.80–1.06	0.250				
			**Coeff**.							
Final absorbance value of the melanization reaction (A_490_)	Linear	Intercept	1.95	0.21	1.54–2.36	**< 0.001**	109	0.160	245.44	7.80
		Host [monarch]	−0.47	0.19	−0.85 – −0.09	**0.016**				
		Parasite [M]	−0.45	0.17	−0.78 – −0.11	**0.010**				
		Parasite [Q]	−0.37	0.17	−0.70 – −0.04	**0.029**				
		Milkweed [Tr]	−0.22	0.14	−0.49 – 0.05	0.117				
		Larval weight	0.57	0.21	0.15–0.99	**0.009**				
			**Coeff**.							
Wing area (mm^2^)	Linear	Intercept	666.11	8.80	648.78–683.44	**< 0.001**	280	0.490	3161.82	7.55
		Host [monarch]	154.53	12.00	130.91–178.15	**< 0.001**				
		Parasite [M]	−11.50	14.59	−40.22 – 17.23	0.431				
		Parasite [Q]	6.02	9.06	−11.82 – 23.87	0.507				
		Milkweed [Tr]	24.78	10.41	4.29–45.27	**0.018**				
		Sex [male]	23.34	8.13	7.34–39.34	**0.004**				
		Host [monarch] × Milkweed [Tr]	−44.46	16.51	−76.96 – −11.97	**0.007**				
			**IRR**							
Adult lifespan (days)	Negative binomial (link = log)	Intercept	12.30	0.63	11.12–13.59	**< 0.001**	285	0.937	1440.06	6.24
		Host [monarch]	1.28	0.11	1.08–1.50	**0.003**				
		Parasite [M]	0.89	0.27	0.46–1.54	0.706				
		Parasite [Q]	0.43	0.03	0.37–0.49	**< 0.001**				
		Milkweed [Tr]	0.95	0.03	0.88–1.02	0.168				
		Sex [male]	1.08	0.04	1.00–1.17	0.052				
		Adult weight	1.19	0.04	1.11–1.28	**< 0.001**				
		Host [monarch] × parasite [M]	0.36	0.11	0.20–0.70	**0.001**				
		Host [monarch] × parasite [Q]	1.18	0.11	0.98–1.42	0.077				
			**IRR**							
Adult lifespan (days) —subset of infected	Quasipoisson (link = log)	Intercept	8.01	0.96	6.33–10.14	**< 0.001**	120	0.930	NA	NA
		Host [monarch]	0.78	0.09	0.62–0.97	**0.024**				
		Parasite [Q]	0.72	0.06	0.60–0.86	**< 0.001**				
		Milkweed [Tr]	1.05	0.06	0.95–1.18	0.34				
		Spore load (log_10_)	0.79	0.05	0.70–0.89	**< 0.001**				
		Sex [male]	1	0.06	0.89–1.13	0.998				
		Adult weight	1.33	0.07	1.20–1.47	**< 0.001**				
		Host [monarch] × spore load (log_10_)	0.79	0.05	0.70–0.89	**< 0.001**				
		Milkweed [Tr] × spore load (log_10_)	1.05	0.05	0.96–1.15	0.268				
		Parasite [Q] × spore load (log_10_)	0.96	0.06	0.85–1.08	0.516				
			**IRR**							
Deformed wings (0/1)	Poisson (link = log)	Intercept	0.02	0.02	0.00–0.12	**< 0.001**	285	0.162	85.382	NA
		Host [monarch]	1.19	1.51	0.08–13.06	0.893				
		Parasite [M]	8.51	9.13	1.11–88.88	**0.046**				
		Parasite [Q]	3.75	3.29	0.71–27.40	0.132				
		Milkweed [Tr]	0.35	0.28	0.05–1.45	0.188				
		Adult weight	0.82	0.52	0.24–2.93	0.757				
		Sex [male]	0.52	0.40	0.10–2.24	0.392				
			**IRR**							
Deformed wings (0/1) —subset of infected	Poisson (link = log)	Intercept	0.01	0.01	0.00–0.12	**0.001**	120	0.445	52.69	NA
		Host [monarch]	2.20	2.84	0.16–32.19	0.544				
		Parasite [Q]	2.86	3.44	0.24–33.37	0.383				
		Milkweed [Tr]	0.20	0.23	0.01–1.30	0.153				
		Spore load (log_10_)	13.59	13.92	2.44–155.97	**0.011**				
		Sex [male]	1.01	0.97	0.14–6.95	0.994				
		Adult weight	0.84	0.59	0.21–3.43	0.809				

Abbreviations: Parasite source: M = monarch parasites, Q = queen parasites. Milkweed host plant species: Sw = low‐cardenolide swamp milkweed, Tr = high‐cardenolide tropical milkweed. Hemocyte type: P = plasmatocytes, S = spheroid cells. Categories used as references are shown inside the square brackets. Estimates: Coeff. = coefficients, OR = odds ratios, IRR = incidence rate ratios. Delta AICc = AICc full model–AICc selected model.

**TABLE A2:** Correlations between adult fitness‐related variables (Spearman's rank correlation coefficient, *r*_*s*_). Values ≥ |0.3| are shown in bold.

				Wing	
	Variable	Weight (g)	Lifespan (days)	Area (mm^2^)	Length (mm)
	Weight (g)	1			
	Lifespan (days)	**0.37**	1		
**Wing**	Area (mm^2^)	**0.9**	**0.25**	1	
	Length (mm)	**0.93**	**0.32**	**0.96**	1

*Note*: Purple used for fitness‐related variables.

**TABLE A3:** Correlations between immune defense variables (Spearman's rank correlation coefficient, *r*_*s*_). Values ≥ |0.3| are shown in bold.

Variable	Final absorbance (A_490_)	Maximum slope (A_490_/read)	Granulocytes (cells/μL)	Plasmatocytes (cells/μL)	Spheroids (cells/μL)	Oenocytoids (cells/μL)	Hemocytes (cells/μL)	Larva weight (g)
Final absorbance of the melanization reaction (A_490_)	1							
Maximum slope of the melanization reaction (A_490_/read)	**0.92**	1						
Granulocytes (cells/μL)	**0.32**	0.22	1					
Plasmatocytes (cells/μL)	0.22	0.19	−0.01	1				
Spheroids (cells/μL)	0.12	0.05	0.06	**−0.26**	1			
Oenocytoids (cells/μL)	−0.14	−0.12	−0.07	−0.03	0.02	1		
Hemocytes (cells/μL)	**0.4**	**0.41**	0.25	**0.31**	−0.09	−0.09	1	
Larva weight (g)	0.13	0.01	0.22	0.17	**−0.23**	−0.05	−0.22	1

*Note*: Green used for immune defense variables.
